# Expert Opinion on the Use of Probiotics in Recurrent Pregnancy Loss

**DOI:** 10.7759/cureus.81056

**Published:** 2025-03-23

**Authors:** Ameet Patki, K Kunjimoideen, Sheetal Sawankar, Rajul Tyagi, Vandana Hegde, Jyoti Budi

**Affiliations:** 1 Obstetrics and Gynecology, Indian Society for Assisted Reproduction (ISAR), Mumbai, IND; 2 Obstetrics and Gynecology, Asian Reproductive Medicine Centre, Kochi, IND; 3 Obstetrics and Gynecology, Avisa IVF and Fertility Center, Mumbai, IND; 4 Obstetrics and Gynecology, Javitri Hospital and Test Tube Baby Centre, Lucknow, IND; 5 Obstetrics and Gynecology, Hegde Fertility, Hyderabad, IND; 6 Obstetrics and Gynecology, Ferty9 Fertility Center, Hyderabad, IND

**Keywords:** lactobacillus, miscarriage, probiotics, recurrent pregnancy loss, reproductive outcomes

## Abstract

Recurrent pregnancy loss (RPL) involves multiple consecutive miscarriages in early pregnancy, affecting a significant number of Indian women and placing substantial physical and emotional stress on expecting couples. This expert consensus aims to highlight probiotics as a promising option for enhancing fertility and supporting successful pregnancy outcomes, offering hope to individuals and couples affected by RPL. A group of fourteen experts with diverse expertise in gynecology, obstetrics, and fertility from across India gathered between June 29 and June 30, 2024. According to the experts, advanced maternal age emerges as an independent risk factor for miscarriage, with increased risks among older Indian women. The major contributors to RPL include thyroid disease and polycystic ovarian disease. Experts emphasize that the vaginal microbiome dysbiosis, characterized by the reduced dominance of *Lactobacilli*, is associated with adverse pregnancy outcome, such as preterm birth, early pregnancy loss, and increased events of RPL. Oral probiotic supplementation, particularly strains like *L. acidophilus* and *L. rhamnosus*, may improve embryo implantation, reduce miscarriage risk, and support pregnancy maintenance. A healthy lifestyle choice and minimal use of antibiotics are important in creating a positive reproductive outcome. The present expert opinion supports the potential benefits of probiotics, particularly *Lactobacillus* species, in managing RPL and improving reproductive outcomes. By promoting a balanced microbiota, reducing inflammation, and modulating immune responses, probiotics may play a critical role in enhancing reproductive success.

## Introduction and background

Probiotics are live microorganisms that, when administered in adequate amounts, confer a health benefit on the host [1]. These beneficial bacteria, predominantly from the genera *Lactobacillus* and *Bifidobacterium* [1], are commonly found in fermented foods and dietary supplements and are naturally present in the human gut. Unlike harmful bacteria that cause diseases, probiotics help maintain a balanced microbiota, which is crucial for overall health. They play a significant role in digestion [2], immunity [3], and even mental health [4] by ensuring the gut remains a stable and healthy environment.

The primary mechanism by which probiotics exert their benefits involves the modulation of the gut microbiota. They enhance the gut barrier function, preventing pathogenic bacteria from colonizing the gut and causing infections. Probiotics also produce antimicrobial substances like bacteriocins and organic acids that inhibit harmful bacteria. Furthermore, they interact with the gut-associated lymphoid tissue (GALT) to stimulate a balanced immune response. By competing for nutrients and adhesion sites, probiotics reduce the colonization and growth of pathogens. Additionally, they help in the production of short-chain fatty acids (SCFAs) like butyrate, propionate, and acetate, which have anti-inflammatory properties and provide energy to colonocytes, thus promoting a healthy gut environment.

Recurrent pregnancy loss (RPL), also referred to as recurrent miscarriage or habitual abortion, is a condition characterized by the occurrence of three or more consecutive pregnancy losses before 20 weeks of gestation [5]. This condition affects about 7% of Indian women trying to conceive and can have profound physical and emotional impacts on affected individuals and couples [6]. The causes of RPL are multifactorial and may include genetic, anatomical, hormonal, immunological, and environmental factors.

The impact of RPL on individuals extends beyond physical health, often leading to significant emotional distress, anxiety, and depression. The repetitive nature of the losses can be particularly devastating, fostering feelings of helplessness and grief. Understanding the underlying causes is crucial for managing and treating RPL. This often requires a comprehensive evaluation involving genetic testing, imaging studies, hormonal assessments, and immunological testing to identify and address the specific factors contributing to the condition.

In recent years, research has begun to explore the potential role of probiotics in managing RPL. The gut microbiota is increasingly recognized for its influence on systemic health, including reproductive health. Probiotics may offer a promising adjunctive treatment by modulating the immune response, reducing inflammation, and improving overall gut health, which in turn could positively impact reproductive outcomes. Although the precise mechanisms and effectiveness of probiotics in RPL management are still under investigation, the potential benefits underscore the importance of maintaining a healthy gut microbiome for reproductive health. This expert consensus seeks to establish probiotics as a viable option for improving fertility and supporting successful pregnancy outcomes, providing hope for individuals and couples affected by RPL.

## Review

Methods

A physical expert meeting was conducted between June 29 and June 30, 2024, to discuss the use of probiotics in women's reproductive health, with a particular focus on RPL. The panel included 14 experts with diverse expertise in gynecology, obstetrics, and fertility from across India. The primary objective was to explore the role of probiotics in enhancing women's reproductive health, including their potential benefits in fertility treatments. Following extensive discussions, key insights from the meeting were compiled into this document. The document was then shared with the experts for review and feedback. Based on their feedback and suggestions, this expert opinion report was prepared and circulated to all experts for final approval.

Etiology of recurrent pregnancy loss

**Figure 1:**
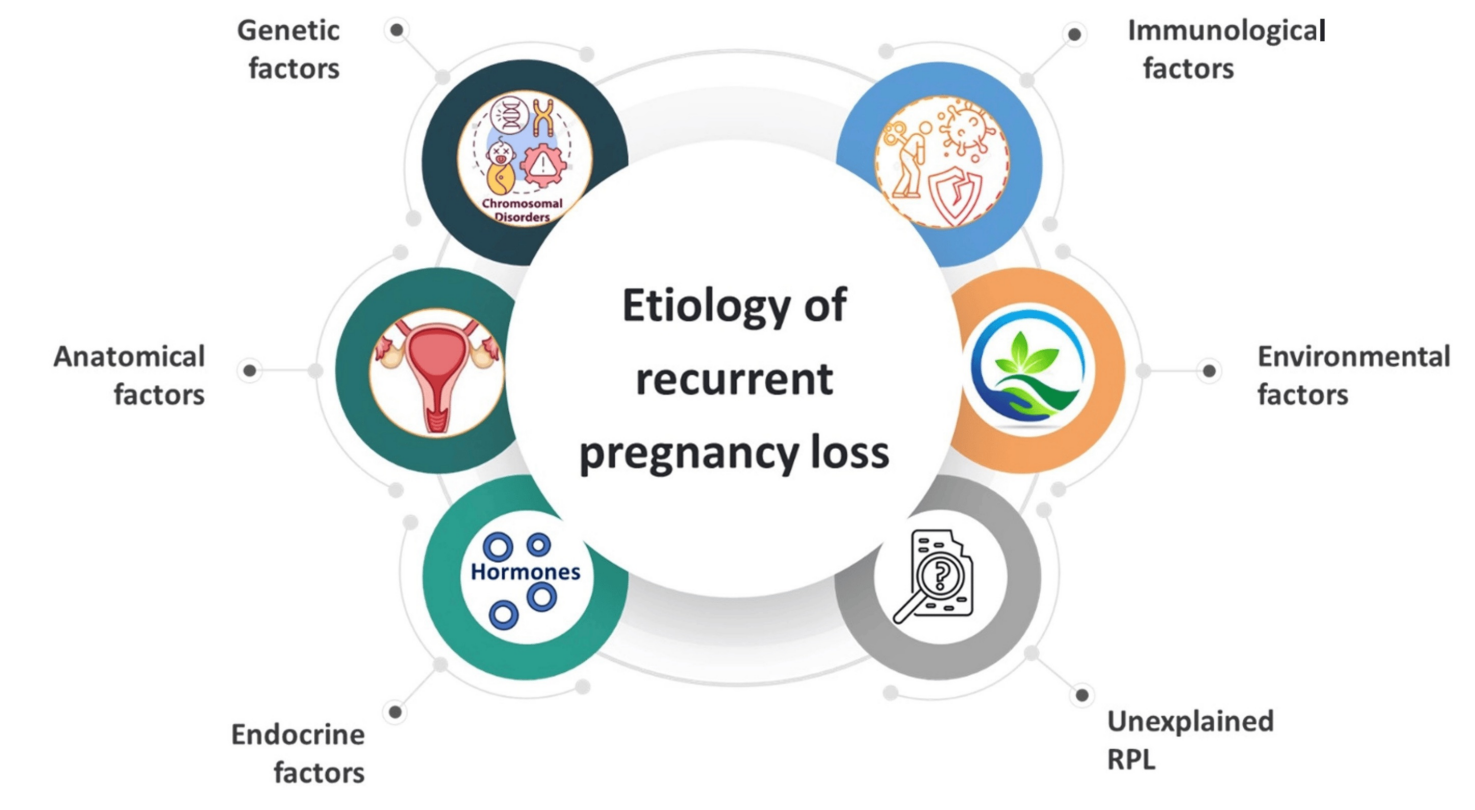
Etiology of recurrent pregnancy loss (RPL) Image created by the authors.

Recurrent pregnancy loss is a complex condition that challenges diagnostic and therapeutic efforts due to various factors. The high baseline rate of spontaneous pregnancy losses, lack of a consistent definition for RPL, limited access to tissues for study, and the generally good prognosis for live birth among RPL patients contribute to the difficulty in establishing clear guidelines. Despite these challenges, some recognized etiologies of RPL include genetic factors, anatomical factors, endocrine factors, immunological factors, infectious factors, and unexplained factors (Figure 1).

Genetic Factors

Genetic factors are implicated in about 2% to 4% of RPL cases [5], often involving balanced structural chromosome rearrangements like reciprocal or Robertsonian translocations. Other genetic anomalies such as chromosomal inversions, insertions, and mosaicism can also contribute. Although less common, single-gene defects, such as those related to cystic fibrosis or sickle cell anemia, may also be involved. Genetic counseling and appropriate evaluation, including parental karyotyping, are essential in cases of RPL associated with chromosomal abnormalities. Depending on the findings, therapies may include in vitro fertilization with preimplantation genetic diagnosis or the use of donor gametes in cases of recurrent aneuploidy [5].

Anatomical Factors

Anatomic abnormalities account for 10% to 15% of RPL cases and are believed to disrupt the vascular supply to the endometrium, leading to abnormal placentation [5]. Congenital uterine anomalies, intrauterine adhesions, and uterine fibroids or polyps are among the potential causes. Congenital uterine anomalies, particularly the uterine septum, are strongly associated with RPL, with some patients experiencing up to a 76% risk of spontaneous pregnancy loss [7]. Other Müllerian anomalies, such as unicornuate, didelphic, and bicornuate uteri, have also been linked to an increased risk of RPL. Diagnostic evaluation typically involves hysteroscopy or hysterosalpingography (HSG), with surgical intervention, such as hysteroscopic resection or myomectomy, considered based on specific findings [7].

Endocrine Factors

Endocrine disorders play a role in about 17% to 20% of RPL cases [8]. Luteal phase defect (LPD), polycystic ovarian syndrome (PCOS), diabetes mellitus, thyroid disease, and hyperprolactinemia are among the implicated conditions. While LPD's role in RPL is controversial, PCOS is often associated with RPL, with insulin resistance and hyperinsulinemia potentially contributing. Poorly controlled diabetes mellitus and untreated hypothyroidism are also linked to increased risks of spontaneous miscarriage. Evaluation should include thyroid-stimulating hormone (TSH) levels and other tests as indicated, with therapy options including insulin-sensitizing agents for RPL associated with PCOS [5].

Immunological Factors

Immunological factors, particularly antiphospholipid antibody syndrome (APS), are recognized causes of RPL. APS is associated with multiple pregnancy complications and requires specific diagnostic criteria, including clinical and laboratory evidence. Once diagnosed, treatment typically involves low-dose aspirin and prophylactic low-molecular-weight heparin, which are safe during pregnancy [5].

Environmental Factors

Environmental exposures often concern patients who experience pregnancy losses, as they may feel responsible or guilty about these events. While research suggests potential links between environmental factors, such as exposure to organic solvents, medications, ionizing radiation, and toxins, and pregnancy loss, these studies are often retrospective and confounded by other factors, making conclusions difficult [9]. Notably, three modifiable exposures, smoking, alcohol, and caffeine, are frequently scrutinized. Maternal alcoholism is consistently linked to higher rates of spontaneous pregnancy loss, though moderate alcohol intake's impact is less clear, with some studies indicating increased risk at higher consumption levels [10-12]. Cigarette smoking, known for nicotine's vasoconstrictive effects, is controversially associated with pregnancy loss, with evidence being inconsistent [12-14]. Similarly, caffeine intake, even at moderate levels, shows a dose-dependent increase in risk for spontaneous pregnancy loss, though the link is weaker for recurrent losses compared to sporadic cases [12,15,16].

Unexplained Recurrent Pregnancy Loss 

Despite extensive evaluation, many RPL cases remain unexplained, making management challenging. Interventions like progesterone therapy and low-dose aspirin have shown potential benefits, but the most effective approach often involves antenatal counseling and psychological support, which significantly improve pregnancy success rates in women with unexplained RPL [17]. Maternal age is another independent risk factor for miscarriage, with higher rates of miscarriage in older women. Indian women aged 31-49 at first birth are more vulnerable to spontaneous miscarriage than younger women. Miscarriage incidence rises from 10% in women aged 20-24 to 51% in women aged 40-44 (Table 1) [6].

**Table 1:** Experts’ comments RPL: recurrent pregnancy loss; PCOD: polycystic ovarian disease

Experts’ comments on factors affecting RPL
Maternal age is an independent risk factor for miscarriage, with older Indian women experiencing higher rates.
Specific conditions such as thyroid disease and PCOD are significant contributors to RPL.
Healthy lifestyle choices, diet, and restricting over-the-counter use of antibiotics without a prescription were emphasized as important lifestyle factors.
Experts strongly recommend the avoidance of smoking, as it has been linked to increased risk of RPL through mechanisms like impaired placental function and vascular damage.
Limit alcohol and caffeine consumption, as excessive intake has been associated with increased miscarriage risk.

Role of the microbiome in pregnancy

Endometrial Microbiome

Recent research has revealed that the endometrium, once thought to be sterile, actually hosts a complex microbial ecosystem. Alterations in this microbiome have been linked to various conditions such as endometriosis, chronic endometritis, dysfunctional menstrual bleeding, endometrial cancer, and infertility [18].

Studies using quantitative polymerase chain reaction (qPCR) have detected bacteria in 95% of endometrial samples from asymptomatic women, with species like *Lactobacillus* *iners*, *Prevotella*, and *Lactobacillus crispatus* being common [19,20]. Further research using 16S rRNA sequencing identified *Lactobacillus spp.* as predominant in the endometrial fluid of healthy women, and a non-*Lactobacillus*-dominated microbiome was associated with lower implantation and live birth rates in infertile women [20]. However, Moreno et al. in their study also questioned the existence of a distinct endometrial microbiome due to similar microbial profiles found in the vaginal and endometrial samples and inconsistencies in findings across different methodologies [20].

The low biomass of the endometrial microbiome presents challenges in analysis, including risks of contamination from vaginal or cervical sources. Thus, strict controls are necessary to ensure accurate results, but as of now, endometrial microbiome testing is not yet applicable in standard clinical practice [18].

Recent studies have highlighted the potential role of the endometrial microbiome in RPL. Liu FT and his colleagues, in their recent study, reported distinct microbial communities in the endometrial tissue and uterine lavage fluid of women with RPL compared to controls. Higher abundances of microbes such as *Acinetobacter, Anaerobacillus, Erysipelothrix, Bacillus,* and *Hydrogenophilus* were noted in the RPL group [21]. Another study identified a predominance of *L. iners* over *L. crispatus *in the endometrial samples from RPL patients. *L. iners* has been linked to endometrial dysbiosis and negative reproductive outcomes [22]. Moreover, this study observed reduced levels of *Lactobacillus spp.* in endometrial samples from RPL patients [22], aligning with previous findings linking lower *Lactobacillus* abundance to poorer reproductive outcomes [20,23]. Additionally, a Chinese study found that increased ureaplasma levels were associated with a higher risk of preterm delivery and pregnancy loss [24]. These findings suggest that disruptions in the endometrial microbiome may contribute to RPL, although further research remains to clarify these associations and their implications for clinical practice.

Vaginal Microbiome

The vaginal microbiome, when healthy, is primarily dominated by *Lactobacillus spp.*, which produce lactic acid and other compounds to maintain an acidic environment that prevents the growth of harmful bacteria [25]. This microbiome can vary significantly between individuals and can fluctuate due to factors such as hormonal changes, unprotected intercourse, or antibiotic use [26-28].

The microbiome has been categorized into different community state types (CSTs) based on dominant bacteria: CST I and CST II are *Lactobacillus*-dominated and generally associated with a healthy state; CST III features *Lactobacillus iners*, which is less acidic and linked to a higher risk of infections; CST IV is marked by a diverse array of anaerobes and is associated with higher risks of bacterial vaginosis and other issues; and CST V, with *Lactobacillus jensenii*, is a transitional state with unclear implications for health [29].

Research on the vaginal microbiome's role in RPL has produced varied results due to differing methodologies and criteria. Studies using light microscopy and bacterial cultures have shown that women with RPL have a significantly higher prevalence of aerobic vaginitis compared to healthy controls [30]. Research has indicated that women with RPL have higher levels of *Atopobium* in their vaginal microbiome compared to those without RPL [31]. Additionally, a relative abundance of *Atopobium* exceeding 0.01% might serve as a potential early marker for predicting first-trimester miscarriages [32]. *Atopobium's* potential to disrupt the vaginal mucosal barrier could trigger inflammation and facilitate the invasion of other bacteria [32].

However, not all research supports these findings. Some studies have found no significant differences in microbial diversity between RPL patients and controls [21,33,34]. Notably, a comprehensive study of 93 pregnancy losses indicated that euploid pregnancy loss is associated with vaginal dysbiosis, though this association was not evident in RPL cases [35].

Overall, these studies highlight the complex and inconsistent relationship between the vaginal microbiome and RPL. While certain bacteria like *Atopobium* are often more prevalent in RPL patients, results vary, underscoring the need for more rigorous research with larger sample sizes and standardized methodologies to better understand the microbiome's impact on RPL and explore potential microbiome-based treatments.

Gut Microbiome

The human gut microbiome, which consists of approximately 100 trillion microbial cells, including bacteria, viruses, and fungi, plays a crucial role in health and disease by influencing nutrition, immunity, and metabolism [36-38]. A balanced gut microbiota is linked to overall health, contrasting with the highly diverse vaginal microbiota. Typically, gut microbiome analysis involves stool samples, rectal swabs, mucosal biopsies, or intestinal fluid, each representing only part of the gastrointestinal tract [39].

Pregnancy induces notable changes in the gut microbiome, impacting pregnancy outcomes [40-42]. Research into the gut microbiome's role in pregnancy loss is limited. One study comparing the fecal microbiome of women with pregnancy losses to controls found reduced microbial diversity and lower abundances of *Prevotellaceae* and *Selenomonas* in the former group [43]. Another study identified that RPL patients with positive antiphospholipid and antinuclear antibodies had higher microbiome diversity, suggesting that an imbalanced microbiome might adversely affect pregnancy outcomes [44].

Microbial profiling is emerging as a promising diagnostic tool for RPL, providing insights into the complex relationship between the microbiome and reproductive health. By analyzing the composition and function of microbial communities in the reproductive tract, researchers can identify dysbiosis, which may contribute to pregnancy loss.

Studies have shown that an imbalance in the vaginal, endometrial, or gut microbiota could influence immune responses, inflammation, and implantation processes, potentially leading to RPL [21,22,24,31,45-48]. Personalized microbial profiling allows for the identification of specific microbial signatures associated with increased risk, enabling tailored interventions such as probiotics or targeted antibiotics to restore microbial balance and improve pregnancy outcomes.

This approach not only enhances the understanding of individual susceptibility to RPL but also offers a pathway for developing novel, non-invasive diagnostic and therapeutic strategies. As research progresses, microbial profiling could become a key component in the clinical management of RPL, providing a more comprehensive view of the factors influencing reproductive success. Table 2 summarizes experts' comments on the role of the microbiome in pregnancy.

**Table 2:** Experts' comments RPL: recurrent pregnancy loss

Experts' comments on the role of the microbiome in pregnancy
Vaginal microbiome dysbiosis, characterized by microbial imbalances, is linked to adverse obstetric outcomes like preterm birth and early pregnancy loss.
Reduced dominance of *Lactobacilli* in the vaginal microbiome increases the risk of pregnancy loss in both spontaneous pregnancies and after in vitro fertilization (IVF).
Dysbiosis in the vaginal flora is associated with higher prevalence in RPL patients, underscoring the critical role of vaginal microflora in reproductive health.
An aberrant vaginal microbiome with reduced *Lactobacilli* dominance should be considered linked to poor reproductive outcomes, such as increased pregnancy loss and lower clinical pregnancy rates following IVF.
Conduct vaginal, endometrial, and gut microbiota assessments to identify dysbiosis and its potential mpact on reproductive health.

Probiotics and the Microbiome

Probiotics can significantly influence the composition and function of the intestinal microbiome through various mechanisms. These beneficial microorganisms produce antimicrobial substances and metabolic compounds that inhibit the growth of harmful bacteria and other pathogens within the gut [49,50]. Additionally, probiotics compete with other intestinal microbes for attachment sites on the intestinal mucosa, thereby reducing the colonization of pathogenic species [51].

*Lactobacillus* strains, in particular, are known to strengthen the integrity of the intestinal barrier. This enhancement helps maintain immune tolerance and reduces bacterial translocation across the gut lining, which can mitigate conditions like gastrointestinal infections, irritable bowel syndrome (IBS), and inflammatory bowel disease (IBD) [52]. Moreover, probiotics can modulate the immune system within the gut, influencing how intestinal epithelial cells and immune cells respond to microbes in the lumen [53].

Probiotics play a critical role in modulating the immune system by interacting with gut-associated lymphoid tissue (GALT). They can influence both the innate and adaptive immune responses. Probiotics promote the maturation of dendritic cells, enhance the activity of natural killer (NK) cells, and increase the production of immunoglobulin A (IgA), which is crucial for mucosal immunity. They also help in balancing pro-inflammatory and anti-inflammatory cytokines, thus promoting a more regulated immune response [54].

Probiotics can help reduce inflammation through several pathways. They produce short-chain fatty acids (SCFAs) like butyrate, which have anti-inflammatory properties and help in maintaining the integrity of the intestinal barrier [24]. Probiotics also inhibit the activation of nuclear factor-kappa B (NF-κB), a key transcription factor involved in the inflammatory response [55]. By promoting the growth of anti-inflammatory bacteria and reducing the presence of pro-inflammatory species, probiotics help to create a balanced gut environment.

Evidence for probiotics in recurrent pregnancy loss

Emerging evidence suggests that probiotics may play a beneficial role in managing RPL and infertility, particularly in the context of assisted reproductive technology (ART). Preliminary studies indicate that certain probiotics might improve pregnancy rates in women undergoing ART, suggesting their potential in preventing RPL [56]. Table 3 summarizes the clinical studies on the role of probiotics in RPL. 

**Table 3:** Clinical studies of probiotics in RPL PPROM: preterm pre-labor rupture of membranes; PROM: pre-labor rupture of membranes; RPL: recurrent pregnancy loss

Author (year)	Condition	Probiotic strain	Outcomes
Fernández et al. (2021) [57]	Reproductive failure (including repetitive abortion and infertility of unknown origin)	*Lactobacillus salivarius* CECT5713	Higher vaginal pH and Nugent scores in women with reproductive failure, lower levels of immune factors (TGF-β1, TGF-β2, VEGF) in women with reproductive failure, 56% successful pregnancy rate with probiotic intervention.
Thanaboonyawat I et al. (2023) [58]	Assisted reproductive technology (ART)	*Lactobacillus* supplementation	No significant difference in biochemical and clinical pregnancy rates between probiotic and control groups, significant decrease in miscarriage rates (9.5% vs. 19.1%), and higher live birth rates in specific subgroups.
Vanda R et al. (2024) [59]	Women undergoing cerclage	Lactobacillus acidophilus, Lactobacillus plantarum, Lactobacillus fermentum, Lactobacillus gasseri	No significant difference in preterm labor rates or mode of delivery, lower rates of PPROM and PROM in the probiotic group, no significant differences in neonatal outcomes.

A study by Fernández et al. [57] compared the cervicovaginal environments of women with reproductive failure (including repetitive abortion and infertility of unknown origin) to those of healthy fertile women. The study found that women with reproductive failure had higher vaginal pH and Nugent scores, along with lower levels of immune factors TGF-β1, TGF-β2, and VEGF. Fertile women had a higher frequency of *Lactobacillus salivarius*, which was nearly absent in one-third of the samples from women with reproductive failure. The intervention with daily oral administration of *L. salivarius *CECT5713 (~9 log10 CFU/day) for up to six months led to a 56% successful pregnancy rate. This probiotic treatment positively influenced key microbiological, biochemical, and immunological parameters in women who became pregnant, suggesting that *L. salivarius* CECT5713 could be an effective option for improving reproductive success in women with reproductive failure.

Prolonged *Lactobacilli *supplementation before embryo transfer has been associated with improved outcomes in ART, potentially shifting the endometrial cavity microbiome towards *Lactobacillus* dominance. This shift may improve embryo survival and implantation rates and reduce miscarriage risk. A randomized controlled trial [58] conducted between August 2019 and May 2021 observed that while biochemical and clinical pregnancy rates were similar between groups receiving *Lactobacillus *supplementation and standard treatment (39.9% vs. 41.8% and 34.2% vs. 31.7%, respectively), the supplementation group experienced a significant decrease in miscarriage rates (9.5% vs. 19.1%, p = 0.02) and higher live birth rates in specific subgroups. For instance, among women with bacterial vaginosis, the live birth rate was higher in the probiotic group (42.31% vs. 26.09%, p = 0.23), and in the blastocyst transfer group, the live birth rate was significantly improved (35.71% vs. 22.22%, p = 0.03). These findings suggest that while probiotics may not improve overall pregnancy rates, they can significantly reduce miscarriage rates and enhance live birth rates in the general population.

Additionally, a recent study [59] assessed the impact of an oral probiotic supplementation (combination of *Lactobacillus acidophilus, Lactobacillus plantarum, Lactobacillus fermentum, Lactobacillus gasseri*) on pregnancy outcomes in women undergoing cerclage, comparing it to a placebo. The study involved 114 participants who were divided to receive either the probiotic or a placebo from the 16th to the 37th week of pregnancy. Results indicated that while there were no significant differences in preterm labor rates or mode of delivery between the groups, the probiotic group had notably lower rates of preterm pre-labor rupture of membranes (PPROM) and pre-labor rupture of membranes (PROM). However, there were no significant differences in neonatal outcomes such as weight, head circumference, height, or Apgar scores. The findings suggest that probiotic supplementation can help reduce specific pregnancy complications like PPROM and PROM.

Preventing preterm birth (PTB) is often more effective than using tocolytic agents to extend gestation. Increasing evidence supports the use of probiotics for PTB prevention. *Lactobacilli*, in particular, may help protect vaginal health by combating pathogens and maintaining vaginal pH, both of which are crucial since vaginal infections are a known risk factor for PTB. Probiotics containing *Lactobacillus rhamnosus *GR1 and *Lactobacillus reuteri *RC14 have been shown to potentially cut the recurrence of vaginal infections and, consequently, the incidence of PTB by 50%. Existing literature suggests that these probiotics are both beneficial and safe when administered during pregnancy, ideally at or before 20 weeks of gestation [60].

While more rigorous clinical trials are needed to fully validate these findings, current evidence supports the potential of probiotics in improving pregnancy outcomes by reducing miscarriage rates, enhancing live birth rates, and managing specific pregnancy complications. Probiotics may offer a valuable adjunctive approach in the management of RPL and infertility. Although some research has indicated beneficial results with probiotic supplementation in women with RPL, not all research supports these findings. For example, a randomized clinical trial examined the impact of intravaginal probiotics prior to frozen embryo transfer in women with recurrent implantation failure (RIF). The study revealed no significant difference in chemical pregnancy rates between the probiotic group (39.02%) and the control group (33.33%) [61].

These varied findings call for more studies in order to clearly establish the effectiveness of probiotics in improving pregnancy outcomes in women with RPL. Current findings confirm that probiotics may improve pregnancy outcomes by decreasing miscarriage rates, boosting live birth rates, and addressing certain pregnancy complications. However, rigorous trials are required to confirm these results. Further, probiotics could serve as a beneficial strategy in managing RPL and infertility (Table 4). 

**Table 4:** Experts’ comments IVF: in vitro fertilization; RPL: recurrent pregnancy loss

Experts' comments on specific probiotic strains for RPL
Long-term *Lactobacillus acidophilus* supplementation before embryo transfer may enhance implantation and embryo survival and reduce miscarriage risk by promoting a *Lactobacillus*-dominant endometrial microbiome.
Lactobacillus strains like *L. acidophilus, L. plantarum, L. fermentum,* and *L. gasseri *play a key role in reducing preterm rupture of membranes (pROM).
Oral *Lactobacillus* supplementation may help prevent miscarriages and support pregnancy.
Probiotic supplementation may improve implantation and pregnancy rates in IVF patients.
*Lactobacillus* supplementation may help reduce first-trimester miscarriage, infertility, and vaginal dysbiosis, improving obstetric outcomes.
Probiotic supplementation with *L. rhamnosus* and *L. salivarius* may benefit couples with recurrent miscarriages linked to immune factors.

Specific probiotic strains

Lactobacillus Species

*Lactobacillus* species play a crucial role in maintaining a healthy vaginal microbiome, which is particularly important during pregnancy. These bacteria produce lactic acid, which lowers the vaginal pH, creating an acidic environment that inhibits the growth of pathogenic microbes. This protective effect helps prevent infections that could lead to adverse pregnancy outcomes, such as preterm birth, miscarriage, and other complications [62].

Studies have shown that *Lactobacillus*-dominant vaginal flora is associated with a lower risk of bacterial vaginosis, a condition linked to preterm labor and other pregnancy-related issues [63]. Additionally, certain *Lactobacillus* strains, such as *Lactobacillus rhamnosus* and *Lactobacillus reuteri,* have been shown to modulate immune responses, reducing inflammation and potentially lowering the risk of pregnancy complications [60].

Bifidobacterium Species

*Bifidobacterium* species are another important group of probiotics with significant benefits during pregnancy. These bacteria are among the first to colonize the human gut and play a pivotal role in maintaining gut health by producing short-chain fatty acids (SCFAs), which provide energy for intestinal cells and support the gut barrier function [64].

In pregnancy, *Bifidobacterium spp.* have been linked to positive outcomes, including effects on the cesarean section rate, birth weight, or gestational age [65]. They also contribute to the development of the neonatal immune system when transferred from mother to baby during birth, providing a foundation for a balanced gut microbiota in the infant [66].

Other Beneficial Strains

Beyond *Lactobacillus* and *Bifidobacterium* species, other probiotic strains have shown potential benefits during pregnancy. *Saccharomyces boulardii*, a non-pathogenic yeast, has been studied for its ability to prevent diarrhea and gastrointestinal disorders, which are sometimes complications during pregnancy [67]. This strain has anti-inflammatory properties and can enhance gut barrier function, potentially reducing the risk of inflammatory-related pregnancy complications.

Additionally, *Streptococcus thermophilus* and *Enterococcus faecium* have been used in combination with other probiotics to improve gut health, enhance nutrient absorption, and modulate the immune system [68]. These strains may help mitigate gastrointestinal discomforts like constipation, which is common during pregnancy, by promoting a healthy digestive system.

Safety and efficacy of probiotics during pregnancy

The safety profile of probiotics during pregnancy is generally favorable, with most studies indicating that they are safe for use. Probiotics are typically well-tolerated, and adverse effects are rare. Clinical trials and observational studies have shown that probiotic supplementation does not pose significant risks to pregnant individuals or their fetuses. For instance, a comprehensive review found no evidence of adverse effects on pregnancy outcomes, including birth weight, preterm birth, or fetal development [65]. The safety of probiotics is further supported by their historical use in various populations, including pregnant women, without significant safety concerns [69].

While probiotics are generally safe, some potential side effects and contraindications may be associated with their use. Common, mild side effects can include gastrointestinal symptoms such as bloating, gas, and mild abdominal discomfort. These effects are usually transient and resolve on their own [70]. Rarely, individuals with compromised immune systems or those with underlying health conditions may experience more serious side effects, such as infections or sepsis, especially with certain probiotic strains [71]. As a precaution, pregnant individuals should consult their healthcare provider before starting any probiotic regimen, particularly if they have pre-existing health issues.

Long-term use of probiotics during pregnancy is generally considered safe, but it is essential to consider certain factors. Prolonged probiotic use may require monitoring for any emerging side effects [72]. Studies have shown that continuous probiotic supplementation does not adversely affect pregnancy outcomes and can be beneficial in maintaining a balanced gut microbiota and preventing complications such as gestational diabetes and preterm birth [65,73]. However, ongoing research is needed to better understand the long-term impacts and optimal duration of probiotic use during pregnancy.

Clinical recommendations for probiotics in pregnancy

The dosage of probiotics is measured in colony-forming units (CFUs), representing the number of viable probiotic cells in a given amount. Probiotic supplements commonly contain between one billion (1 × 10⁹) and 10 billion CFUs per dose [74]. Generally, a probiotic dose ranging from 10⁶ to 10⁸ CFUs per gram or higher, up to 10⁸ to 10¹⁰ CFUs per gram, has been shown to be effective in most clinical studies. However, according to the World Gastroenterology Organization, there is no universally recommended dosage for probiotics [75]. Some probiotics may exert positive effects even at lower dosages, while others may require higher dosages to achieve the desired therapeutic outcomes. Moreover, the impact of each probiotic on specific health conditions may differ, necessitating tailored dosages based on individual patient needs and the specific clinical scenario.

In clinical settings, strain designation is crucial, as it links specific strains to clinical benefits. For example, certain strains of *Lactobacillus* or *Bifidobacterium* have been associated with preventing specific types of diarrhea or improving gastrointestinal health. Some strains may possess unique properties that provide distinct medical benefits, while emerging evidence suggests that certain mechanisms of probiotic action are shared across different strains, species, and genera [74]. Table 5 summarizes expert insights on clinical recommendations for probiotics in pregnancy.

**Table 5:** Experts’ comments IVF: in vitro fertilization; RPL: recurrent pregnancy loss; CFU: colony-forming units

Expert insights on clinical recommendations for probiotics in pregnancy
Prolonged supplementation of *Lactobacillus acidophilus* before embryo transfer has the potential to shift the endometrial microbiome towards *Lactobacillus* dominance, potentially improving embryo survival, implantation rates, and reducing miscarriage risk.
Healthy lifestyle choices, diet, and restricting over-the-counter use of antibiotics without a prescription were emphasized as important lifestyle factors.
*Lactobacillus* probiotic strains, including L*. acidophilus, L. plantarum, L. fermentum,* and *L. gasseri,* were noted for their significant role in reducing preterm rupture of membranes (pROMs).
Oral *Lactobacillus* supplementation should be considered to potentially prevent miscarriages and support pregnancy maintenance.
Probiotic supplementation should be recommended to enhance implantation and pregnancy rates among patients undergoing IVF.
*Lactobacillus* supplementation should be recommended to potentially reduce first-trimester miscarriage, infertility, and vaginal microbiome dysbiosis to improve obstetric outcomes.
Probiotic supplementation, including *L. rhamnosus *and *L. salivarius*, should be considered for couples experiencing habitual abortion attributed to immunologic factors.
For RPL, the recommended dosage of probiotic supplementation typically falls within the range of 10 to 20 billion CFU per day.
Probiotic supplementation is often suggested for a duration of one to three months before conception or continued throughout early pregnancy to help restore vaginal and gut microbiota balance, reduce inflammation, and support immune regulation.
As protocols may vary, it’s crucial to consult with a healthcare professional to determine the most appropriate dosage and duration based on individual circumstances.

## Conclusions

This expert consensus highlights the importance of a comprehensive and individualized approach to managing RPL, integrating advanced diagnostic tools and tailored lifestyle interventions to optimize reproductive outcomes. Emerging evidence highlights the potential benefits of probiotics in managing RPL and improving reproductive outcomes. Probiotics, particularly those containing *Lactobacillus* species, play a critical role in maintaining a balanced microbiota, which may be beneficial in reducing inflammation and modulating immune responses relevant to RPL. Clinical studies suggest that probiotics can enhance reproductive success, particularly in ART, by improving pregnancy rates and reducing miscarriage rates.

While current findings are encouraging, more rigorous and large-scale studies are needed to confirm the efficacy of probiotics in RPL management and to better understand the underlying mechanisms. Although probiotics have shown significant potential in improving various health outcomes, continued research should focus on standardizing probiotic strains, dosages, and treatment durations to establish definitive guidelines. Given the variability in strain-specific benefits and the absence of universally recommended dosages, it is essential to tailor probiotic use to individual patient needs.
